# Updated cholangiocarcinoma incidence trends and projections in Thailand by region based on data from four population-based cancer registries

**DOI:** 10.1016/j.lansea.2025.100569

**Published:** 2025-03-29

**Authors:** Oraya Sahat, Surichai Bilheem, Apiradee Lim, Siriporn Kamsa-ard, Apiporn Thinkhamrop Suwannatrai, Surin Uadrang, Atit Leklob, Wasan Chansaard, Nithima Sriket, Chalongpon Santong, Karnchana Daoprasert, Supot Kamsa-ard

**Affiliations:** a Student of Doctor of Public Health Program, Faculty of Public Health, Khon Kaen University, Khon Kaen, Thailand; b Sirindhorn College of Public Health Yala, Faculty of Public Health and Allied Health Sciences, Praboromarajchanok Institute, Yala, Thailand; c Department of Science in Mathematics with Computer Science, Faculty of Science and Technology, Prince of Songkhla University Pattani Campus, Pattani, Thailand; d Department of Epidemiology and Biostatistics, Faculty of Public Health, Khon Kaen University, Khon Kaen, Thailand; e Department of Parasitology, Faculty of Medicine, Khon Kaen University, Khon Kaen, Thailand; f Lop Buri Cancer Hospital, Lop Buri, Thailand; g Surat Thani Cancer Hospital, Surat Thani, Thailand; h Cancer Unit, Srinagarind Hospital, Faculty of Medicine, Khon Kaen University, Khon Kaen, Thailand; i Lampang Cancer Hospital, Lampang, Thailand

**Keywords:** Cholangiocarcinoma, Trends, Incidence, Projections, Cancer registies

## Abstract

**Background:**

Cholangiocarcinoma (CCA) is a significant health concern in Thailand, as the age-standardized rates (ASR) and other trends fluctuate across different regions. However, comprehensive national estimates are lacking. This study examined the Thai ASR of CCA trends from 2012 to 2021 and projected the incidence rates to 2026.

**Methods:**

This retrospective cohort analysis examined 6379 CCA cases from population-based cancer registries (PBCRs) in the northern, central, northeastern, and southern regions for the time period January 1, 2012, to December 31, 2021. The Joinpoint, age-period-cohort, and Nordpred models were used to assess CCA incidence trends and predictions.

**Findings:**

CCA incidence trends in Thailand showed a decrease, with an average annual percentage change (AAPC) of −7.20% (95% CI: −11.04 to −3.19) for males, and −5.81% (95% CI: −10.81 to −0.54) for females. The projected incidence rate per 100,000 person-years for 2026 varied slightly according to the model: Joinpoint (males: 6.1, females: 3.4), age-period-cohort (males: 6.0, females: 3.3), and Nordpred (males: 5.5, females: 3.4). Regional analyses revealed decreasing trends in the northern and northeastern regions, with 2026 projections indicating further declines exceeding the 10-year trends. Owing to the small sample size, trends in the central and southern regions could not be determined.

**Interpretation:**

Thailand's CCA rate has generally decreased but varies geographically; the northern and northeastern regions remain at high risk. To minimize CCA nationally, initiatives should be maintained, new risk factors explored, diagnostics improved, and regional variances addressed.

**Funding:**

The Graduate School of Khon Kaen University.

**Research in context**

**Evidence before this study**

Thailand has been recognized as a global epicenter of cholangiocarcinoma (CCA), particularly in the northeastern region. Between 1989 and 2018, the respective rate for males and females was 36.1 and 14.4 per 100,000 person-years. The respective projected incidence rate of bile duct cancer in males and females from 2019 to 2028 decreased to 7.6 and 3.6 per 100,000 person-years. Research has linked elevated incidence of *Opisthorchis viverrini* (*O. viverrini*) infection with CCA, emphasizing the connection between parasitic exposure, diet, and cancer development.

**Added value of this study**

This is the first study to investigate the incidence of CCA in Thailand using a comprehensive nationwide analysis. This is to help analyze trends in CCA incidence and to include long-term data for ten years and five years of projections. This study offers insight into CCA distribution and trends using sophisticated statistical modeling techniques, including Joinpoint regression, age-period-cohort, and Nordpred models. The methodology introduced rigorous data quality assessment indicators, utilizing detailed International Classification of Diseases for Oncology (ICD-O-3) codes and implementing advanced techniques to minimize projection errors.

**Implications of all the available evidence**

This CCA study provides comprehensive insights into cancer incidence in Thailand by employing advanced statistical modeling across four regional registries. The research reveals significant geographical variations in cholangiocarcinoma rates, highlighting disparities between Northern/Northeastern and Central/Southern regions. This research provides critical guidance for targeted public health interventions and future epidemiological investigations by exposing localized risk factors and demonstrating methodological innovations.

## Introduction

The age-standardized rates (ASRs) of cholangiocarcinoma (CCA) vary widely between regions and nations. Intrahepatic (ICC) and extrahepatic cholangiocarcinoma (ECC) rates are rising worldwide, with the highest rates in Asian countries, including South Korea, Thailand, and Japan.^1^ ICC mortality has increased in the USA, the UK, and Australia, whereas ECC mortality has declined in most European and Australasian nations but increased in the Americas.^2^ Over the past 12 years, CCA incidence has increased in the US, especially among Hispanics and those with diabetes and obesity.^3^ These data show the complicated worldwide epidemiological picture of CCA, highlighting the need for more studies to understand the causes.

The worldwide CCA burden is the highest in Thailand, Laos PDRs, Vietnam, and Cambodia-countries around the Mekong River due to hepatic fluke infestation.^4^ These regional patterns are explained by multiple causes, including metabolic effects, viral etiologies, and improved cancer identification techniques.^5^^,^^6^ The persistence of CCA despite control efforts highlights the need for risk factor research and novel diagnostic techniques and therapeutics to address CCA genetic heterogeneity and enhance patient outcomes.^7^

Thailand ranks among the countries with the highest CCA rates. Within Thailand, the ASR of CCA is highest in males, especially in the Northeast and North, making this a unique case study.^8^, ^9^, ^10^, ^11^ Northeastern Thailand has the highest CCA rate of 85 per 100,000 person-years, primarily due to liver fluke infection.^12^^,^^13^ The Southern region had the lowest incidence at 5.7 per 100,000 person-years, while the Northern and Central regions had 14.5 per 100,000 person-years.^6^

The regional CCA incidence in Thailand shows a complicated interaction of environmental, nutritional, and parasite variables.^14^, ^15^, ^16^ Several interrelated variables explain the variance in the incidence. The northeastern region has the highest risk, with a greater prevalence of *Opisthorchis viverrini (O. viverrini)*.^17^ Regional diets, particularly those that include raw or undercooked freshwater fish with liver fluke metacercaria, increase this risk.^18^ Specific polymorphisms may increase the vulnerability of different populations.^19^ Regional variations in chemical and toxin exposures exacerbate these discrepancies.^20^ Discrepancies in healthcare access and early detection programs can further affect incidence rates.^21^ Understanding this complex web of determinants is essential for creating targeted public health strategies to reduce CCA in Thailand.

Previous studies lacked long-term incidence rate data, and recent reports were incomplete, with only some regions being recorded. Regional data on incidence rates and predictions are also limited. Thus, this study examined CCA trends from 2012 to 2021 and projected ASR trends through 2026 in Thailand. This extensive analysis of Thai CCA trends aims to contribute to national public health initiatives for CCA prevention and management, particularly in high-risk areas with increasing incidence. Our ultimate objective was to reduce the national CCA rate.

## Methods

### Study design, data sources, and data collected

This retrospective cohort analysis examined CCA cases from four population-based cancer registries (PBCRs). The study covered four regions: northern (Lampang Cancer Hospital), central (Lop Buri Cancer Hospital), northeastern (Khon Kean Provincial Cancer Registry, Srinagarind Hospital, Faculty of Medicine, Khon Kaen University), and southern (Surat Thani Cancer Hospital) from January 1, 2012, to December 31, 2021.^8^ The International Classification of Diseases for Oncology, 3rd Edition (ICD-O-3) codes C22.1 (intrahepatic bile duct), C24.0 (extrahepatic), C24.8 (overlapping biliary lesion), and C24.9 (Biliary tract, NOS) were used to identify cases.^22^^,^^23^

The data collected included age at diagnosis, sex, date of birth, year of diagnosis, basis of diagnosis (death certificate only (DCO), history and physical examination, endoscopy and radiology, surgery and autopsy (no histology), specified biochemical/immunological tests, cytology of hematology, histology of metastasis, and histology of primary). Our study analyzed data from all patients with CCA included in the PBCRs with complete information available. No information was missing when examining the cancer registry data, as this was the standard protocol for recording data in the PBCRs.

A log-linear function was used to assess the population denominators for the incidence rates. For 2012–2026, this approach interpolates between two successive population census sizes stratified by sex and 18 age groups (0–4 to 85+). The National Economic and Social Development Board offered these estimates.^24^

Two essential factors were used to assess data quality using the PBCR diagnostic codes. The first indicator measured diagnostic validity using the death certificate-only case percentage (DCO%). A low DCO% indicates high-quality data with comprehensive incidence notification, whereas a high DCO% indicates potentially less reliable CCA diagnoses. The second indicator, microscopically confirmed cases (MV%), assessed the diagnostic information, quality, and completeness. A high MV% indicates a valid CCA diagnosis, whereas a low MV% suggests that insufficient pathology reporting might be the cause of diagnostic errors. These indications were applied according to the NCI^8^ and IARC^25^ criteria.

### Statistical analysis

The ASR was calculated using CCA data and population denominators and standardized using the Segi world standard population estimates.^26^ The results are presented as cases per 100,000 individuals with 95% confidence intervals (CIs). These ASRs form the basis for all trend estimates and incidence projections.

Trend analysis employed Joinpoint regression to identify significant changes in CCA incidence over time. This method analyzes trends in specific sex and age groups by computing the annual percentage change (APC) and average annual percentage change (AAPC) over the study period.^27^

The incidence of CCA was projected using the Joinpoint, age-period cohort, and Nordpred models. Each model uses distinct methods to analyze and predict future trends. All models used a cut-trend to avoid overestimating the long-term projections. At 5-year intervals, this strategy reduced the linear drift by 0, 25, 50, and 75 percent. The incidence value was changed to minimize drift by 8% annually beyond the observation period; however, but the projected rates of the second future period were unaffected. Geometric dampening reduced between 2022 and 2026 by 21.6%.^28^, ^29^, ^30^

First, Joinpoint regression captures trend changes using segmented line regression. This method represents the CCA ASR as a function of time. The model utilized change points (Joinpoint) and their estimated number, with a maximum of two Joinpoints and a minimum of two observations from each end of the data. This approach identifies significant changes over time.^31^, ^32^, ^33^

Second, the age-period cohort model examined CCA incidence rates according to age, year of diagnosis (period), and year of birth. This model addresses age-period-cohort identifiability using log-linear regression.^29^^,^^30^ The investigation used Akaike Information Criterion-based, two-effect models (AP-C and AC-P), using the reference years 2012 and 1960. The models were fitted to the CCA data by sex using Epi in R.^34^^,^^35^ Natural splines reduce random variation.

Third, Nordpred used the age-period-cohort model to isolate the influence of age, period, and birth cohort on CCA incidence.^36^ All observation periods had a lower age restriction of 25 years, and this model extended trends from the observed data to 5-year intervals. Overestimation was avoided by powering the multiplicative model using five different functions. The projection uses a geometric cut trend and a linear drift approach, similar to the age-period-cohort model. One-year estimates were derived from five-year estimates using linear interpolation.^36^^,^^37^

Data analyses were conducted using a comprehensive software approach. R software version 4.2.1 (R Core Team)^38^ and R Studio version 1.4.1 (R)^39^ was used as the primary platforms for data management and statistical analyses. The Joinpoint regression program version 5.0.1^40^ was employed for trend analysis by calculating the APC and AAPC. For CCA incidence projections, three models–Joinpoint, age-period-cohort, and Nordpred–were implemented in R. This integrated approach allowed for a robust trend analysis and future projections of CCA incidence.

### Ethical considerations

The Human Research Ethics Committee of Lampang Cancer Hospital (No. 10/2567), Lop Buri Cancer Hospital (No. LEC 6647), Khon Kaen University (No. HE671027), and Surat Thani Cancer Hospital (No. SCH_EC_01/2567) reviewed and approved the research proposal.

### Role of the funding source

The authors have no financial or non-financial interests to disclose, and the funding source was not involved in this study.

## Results

### CCA incidence nationwide and in four regions

The 4 Thai PBCRs recorded 6379 CCA cases between 2012 and 2021. The mean diagnostic age was 65.5 years for males (SD = 10.8) and 67.3 years for females (SD = 11.4), with the majority of cases concentrated in the 55–74 age range (3908 cases; 61.3%). The age distribution revealed a gradual increase in CCA incidence from ages 40–54 (858 cases; 13.4%), peaking in the 60–74 age range (3108 cases; 48.7%) for both sexes. Notably, very few cases were observed in individuals under 40 (69 cases; 1.1%).

The registry data demonstrated high-quality reporting through low DCO percentages of 1.1% for males and 0.9% for females. The diagnostic methods primarily relied on endoscopy and radiology (69.8% for males, 66.7% for females), supplemented by histological examination of primary sites (17.5% for males, 18.6% for females). The difficulty of microscopic diagnosis of CCA was demonstrated by the 21.6% male and 23.1% female morphological verification rates (Table 1).

**Table 1:** Characteristics of study participants at recruitment by sex in Thailand from 2012 to 2021.

Characteristic	Males		Females	
	Number (n = 4075)	%	Number (n = 2304)	%
**Diagnosis period (year)**				
2012	509	12.5	263	11.4
2013	459	11.2	233	10.1
2014	415	10.2	284	12.3
2015	436	10.7	233	10.1
2016	428	10.5	229	10.0
2017	435	10.7	238	10.3
2018	386	9.5	203	8.8
2019	339	8.3	211	9.2
2020	378	9.3	233	10.1
2021	290	7.1	177	7.7
Mean (Standard deviation)	407.5 (62.2)		230.4 (29.8)	
Median (Minimum: Maximum)	421.5 (290: 509)		233.0 (177: 284)	
**Age at diagnosis (year)**				
15–19	2	0.1	–	–
20–24	1	0.1	5	0.2
25–29	3	0.1	4	0.2
30–34	9	0.2	3	0.1
35–39	29	0.7	13	0.6
40–44	85	2.1	36	1.6
45–49	181	4.4	92	4.0
50–54	323	7.9	141	6.1
55–59	531	13.0	269	11.7
60–64	688	16.9	329	14.3
65–69	678	16.6	386	16.7
70–74	660	16.2	367	15.9
75–79	489	12.0	318	13.8
80–84	282	6.9	224	9.7
85+	114	2.8	117	5.1
Mean (Standard deviation)	65.5 (10.8)		67.3 (11.4)	
Median (Minimum: Maximum)	66 (19: 98)		68 (20: 97)	
**Basis of diagnosis**				
Death certificate only (DCO)	43	1.0	22	1.0
History and physical examination	275	6.7	189	8.2
Endoscopy and radiology	2844	69.8	1536	66.7
Surgery and autopsy (no histology)	13	0.3	12	0.5
Specific biochemical/immunological tests	19	0.5	13	0.6
Cytology of hematology	52	1.3	40	1.7
Histology of metastasis	117	2.9	63	2.7
Histology of primary	712	17.5	429	18.6
**Regions**				
Overall	4075	63.9	2304	36.1
Northern	1060	62.5	635	37.5
Central	383	61.4	241	38.6
Northeastern	2537	65.1	1361	34.9
Southern	95	58.6	67	41.4

n, number of CCA cases.

The overall ASR of CCA between 2012 and 2021 was 12.5 per 100,000 person-years in males (95% CI: 12.1–12.9), 5.9 in females (95% CI: 5.6–6.1), and 8.9 in both sexes (95% CI: 8.7–9.2). Additionally, the ASR in each region was higher in males than females. The northeastern region had the highest ASR (males: ASR = 19.2 per 100,000 person-years, 95% CI: 18.4–19.9; females: ASR = 8.5 per 100,000 person-years, 95% CI: 8.0–8.9) compared to the other regions (Table 2).

**Table 2:** ASR per 100,000 person-year of CCA by year of diagnosis in national and four regions between 2012 and 2021.

n, number of CCA cases. CR, crude rate. ASR, age-standardized rate. CI, confidence interval.

### Trends analysis

**Fig. 1:**
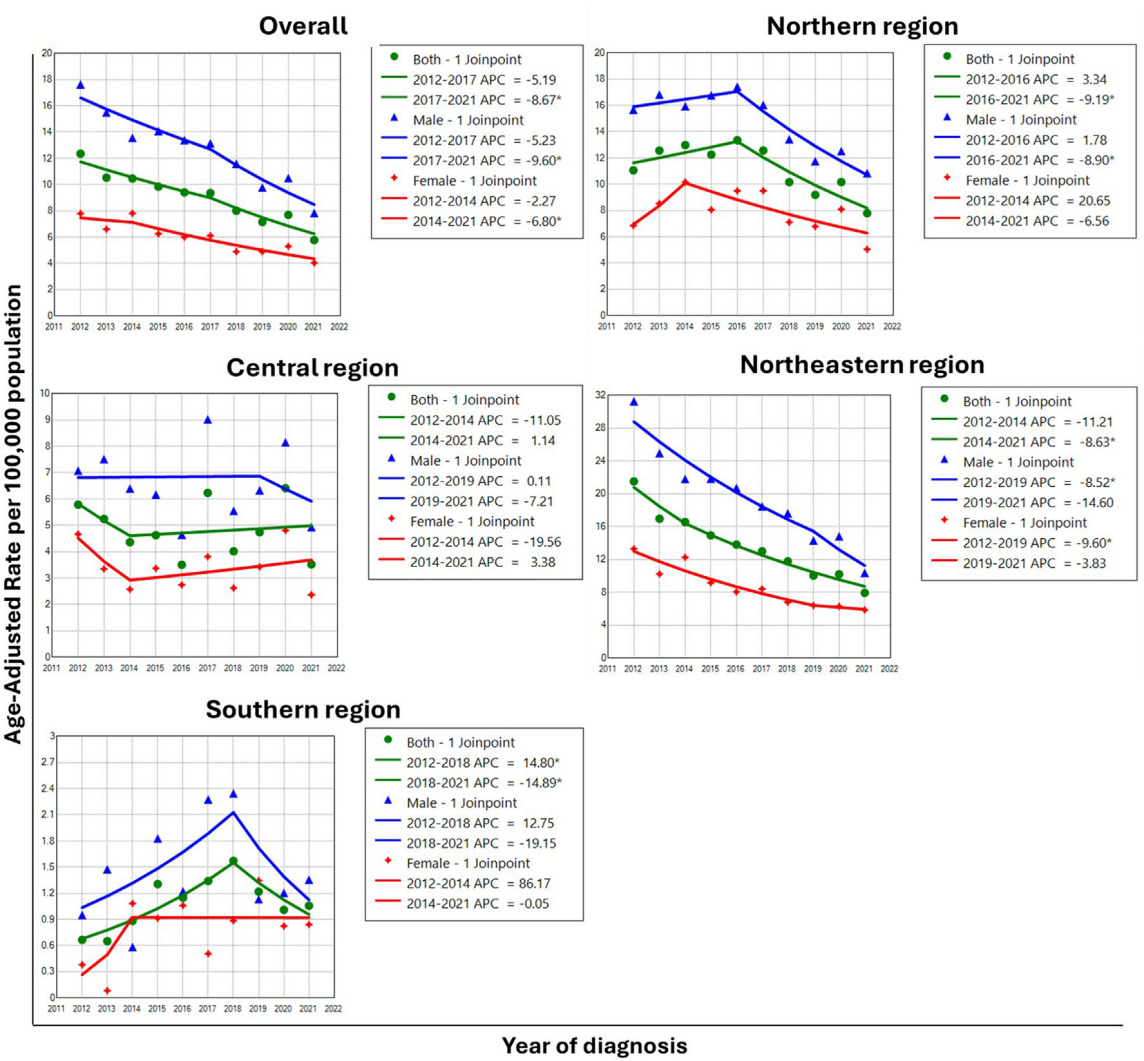
**Joinpoint regression trends in the ASR of CCA by sexes between 2012 and 2021 in the national and four regions**. APC, Annual Percent Change. ∗. Indicates that the APC significantly differs from zero at alpha = 0.05.

Table 3 and Fig. 1 show the Joinpoint model combining all ages and sexes and the ASR curve for CCA incidence. There were two national trend periods, with the results showing that the first trend decreased (male: APC = −5.23%, 95% CI: −10.75 to 0.63; female: APC = −2.27, 95% CI: −26.62 to 30.16), and the second trend decreased (male: APC = −9.60%, 95% CI: −18.16 to −0.14; female: APC = −6.80, 95% CI: −10.60 to −2.84).

**Table 3:** The APC in incidence rate of CCA in four regions between 2012 and 2021.

Regions	Trend-1			Trend-2			AAPC	95% CI
	Periods	APC	95% CI	Periods	APC	95% CI		
**Overall**								
Males	2012–2017	−5.23	−10.75 to 0.63	2017–2021	−9.60	−18.16 to −0.14	−7.20	−11.04 to −3.19
Females	2012–2014	−2.27	−26.62 to 30.16	2014–2021	−6.80	−10.60 to −2.84	−5.81	−10.81 to −0.54
Both	2012–2017	−5.19	−10.29 to 0.20	2017–2021	−8.67	−16.57 to −0.03	−6.75	−10.28 to −3.09
**Northern**								
Males	2012–2016	1.78	−1.82 to 9.37	2016–2021	−8.90	−14.03 to −6.41	−4.30	−6.01 to −2.62
Females	2012–2014	20.65	−5.95 to 57.02	2014–2021	−6.56	−29.08 to 1.96	−1.10	−7.70 to 4.11
Both	2012–2016	3.34	−2.68 to 27.93	2016–2021	−9.19	−27.66 to −5.14	−3.82	−8.13 to 0.19
**Central**								
Males	2012–2019	0.11	−10.45 to 11.92	2019–2021	−7.21	−59.04 to 110.20	−1.56	−15.57 to 14.77
Females	2012–2014	−19.56	−69.99 to 115.68	2014–2021	3.38	−10.17 to 18.98	−2.22	−18.88 to 17.85
Both	2012–2014	−11.05	−66.46 to 135.88	2014–2021	1.14	−11.91 to 16.12	−1.71	−18.26 to 18.20
**Northeastern**								
Males	2012–2019	−8.52	−11.51 to −5.44	2019–2021	−14.60	−37.72 to 17.10	−9.91	−14.90 to −4.63
Females	2012–2019	−9.60	−14.24 to −4.71	2019–2021	−3.83	−39.53 to 52.94	−8.35	−15.78 to −0.26
Both	2012–2014	−11.21	−24.21 to 4.02	2014–2021	−8.63	−10.94 to −6.25	−9.21	−11.96 to −6.36
**Southern**								
Males	2012–2018	12.75	−7.60 to 37.58	2018–2021	−19.15	−56.13 to 48.99	0.92	−16.16 to 21.48
Females	2012–2014	86.17	−86.49 to 258.83	2014–2021	−0.05	−14.98 to 17.51	14.77	−27.17 to 80.84
Both	2012–2018	14.80	9.28–28.66	2018–2021	−14.89	−31.03 to −3.12	3.91	0.24–8.56

APC, Annual Percent Change. AAPC, Average Annual Percent Change. CI, Confidence interval.

Most of the second trend models showed a decrease in both sexes in the northern, northeastern, and southern regions, as well as in males in the central regions. In contrast, the trend in the central region increased for females. The overall trend models showed a decrease from 2012 to 2021, with an AAPC of −6.75% (95% CI: −10.28 to −3.09). When analyzing all age groups and sexes, each region demonstrated decreasing incidence trends in the northern and northeastern regions of the country. Owing to the small number of samples, clear trends were not discernible for the central and southern regions (Table 3, Fig. 1).

### Trend projections

**Fig. 2:**
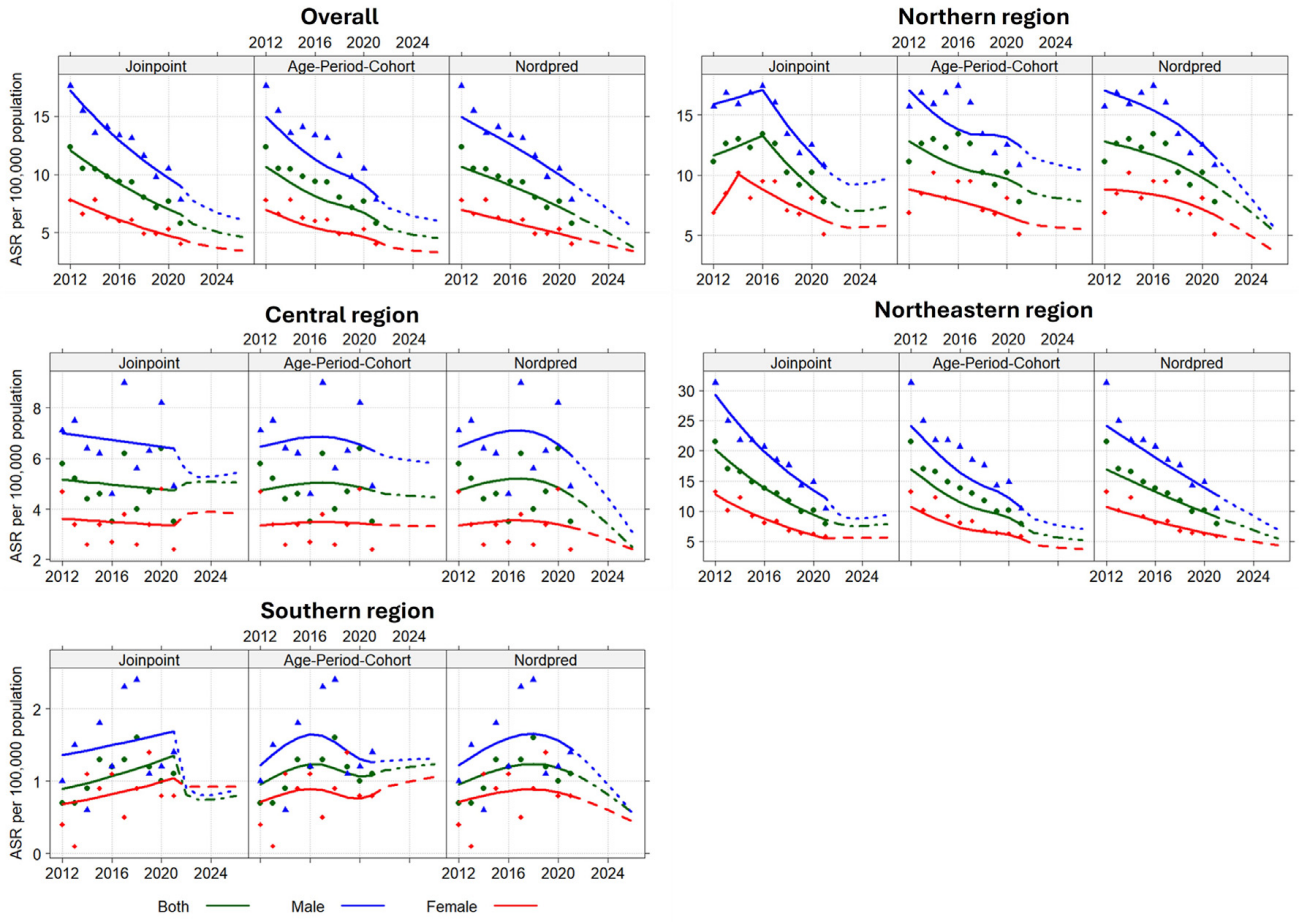
**Shows the ASR trend projections of CCA to 2026 in the national and four regions using the Joinpoint, age-period-cohort, and Nordpred models**. ASR, age-standardized rate.

The national ASR per 100,000 person-years will decrease in all models by 2026. Using the Joinpoint model, the respective males and females ASR was 6.1 and 3.4. The AC-P project model will decline to 6.0 for males and 3.3 for females. The Nordpred incidence-rate-predicted model dropped to 5.5 males and 3.4 females (Fig. 2).

The model projects per 100,000 person-years to 2026 in each region show a decrease compared to the 2012–2021 rates. The respective male; female value for the Joinpoint, AC-P, and Nordpred model in the northern region was (m 9.6; f 5.8), (m 10.5; f 5.5), and (m 5.5; f 3.6). The respective male-to-female values for the Joinpoint, AC-P, and Nordpred models for the central region was (males = 5.4), (males = 5.8; females = 3.3), and (males = 3.1; females = 2.4). The respective male vs. female for the Joinpoint, AC-P, and Nordpred model for the northeastern region was 9.4 vs. 5.7, 7.0 vs. 3.7, and 6.9 vs. 4.4. In the southern region, the males vs. females for the Joinpoint and Nordpred model was 0.9 vs. 0.9 and 0.5 vs. 0.4. In contrast, the projection models to 2026 in the central region, such as the Joinpoint model (females = 3.8) and the AC-P model in the southern region (males = 1.3; females = 1.1), compared with the trends from 2012 to 2021 found that increases (Fig. 2).

## Discussion

The ASR of CCA in Thailand decreased from 2012 to 2021, and all three models predicted additional decreases by 2026. The first national-level research on CCA trends in Thailand provides essential insights into regional variance and long-term tendencies. This study was the most recent national retrospective examination of CCA in Thailand.

The northern and northeastern regions of Thailand have continuously declining CCA incidence. In contrast, the central and southern regions showed different patterns. However, due to the small sample populations in these regions, we cannot draw definitive conclusions about the trends. These geographical differences demonstrate how environmental, nutritional, and parasite variables affect CCA development. Due to the elevated CCA rates linked to *O. viverrini* infection, earlier research has concentrated on northeastern provinces or areas.^14^, ^15^, ^16^

Traditional consumption of uncooked or undercooked cyprinoid fish, which may contain *O. viverrini* larvae, may explain these regional variations. Residents in the southern region rarely consume such dishes, whereas those in the northeastern region commonly do. The northeast has a 34.6% liver fluke infection rates, followed by the central (6.34%), northern (5.59%), and southern (0.01%) regions.^18^^,^^41^^,^^42^ Despite the absence of liver fluke infections in the south, CCA cases were found, perhaps due to cirrhosis and hepatitis B or C.^43^^,^^44^ Population migration and exposure to other risk factors such as nitrosamines may also account for these differences.^15^^,^^44^^,^^45^ Regional variation in *O. viverrini* infection rates parallels the incidence of CCA in Thailand, firmly linking the two.

Our findings corroborate the Khon Kaen Province research (1989–2018)^46^^,^^47^ and the 2008–2012 nationwide ICC trend analysis.^5^ Our findings contrast those from Taiwan and the US, where the CCA incidence is increasing. Cholelithiasis, obesity, and hepatitis B or C are more frequent in those countries than in Thailand,^48^, ^49^, ^50^ whereas *O. viverrini* infection is the primary in Thailand.^15^^,^^18^^,^^51^^,^^52^ Our rates were lower than those reported by GLOBOCAN,^2^ the National Cancer Institute in Thailand (NCI),^8^, ^9^, ^10^ and the Global Burden of Cancer^6^^,^^53^^,^^54^ because these reports included liver and bile duct cancer together.

Practical and effective actions of the Thai Ministry of Public Health have lowered CCA rates. Interventions have targeted *O. viverrini* infection, the primary CCA risk factor.^15^^,^^18^^,^^51^^,^^52^ Pesticide use was not associated with CCA.^55^ After the first autopsy-confirmed *O. viverrini* infection associated with CCA in 1950, the Department of Health established the Intestinal Helminthiasis Control Unit to diagnose and treat intestinal parasites and *O. viverrini*. Since 1987, control methods have encompassed recommendations for regular stool testing for metacercaria, anthelminthic therapy, cooking fish before consumption, and promoting hygienic practices.^56^, ^57^, ^58^ Animal studies have demonstrated that repeated *O. viverrini* infections increase CCA risk, with one initial infection and two to three subsequent reinfections, significantly increasing the likelihood of cancer development. These repeated infections can lead to cumulative cellular damage through oxidative and nitrosative stress, potentially explaining the increased risk of cancer. Frequent praziquantel treatments are thus a marker of repeated infections rather than a direct cause of cellular damage.^45^, ^59^, ^60^, ^61^

The National Opisthorchiasis Control Program has established comprehensive guidelines for screening and prevention. These guidelines advise liver fluke screening for those aged 15 years and older and CCA screening for those ages 40 years and older with a history of *O. viverrini* infection. Individuals who have previously taken praziquantel to treat liver flukes and those who have consumed raw white-scaled fish are at a higher risk. Examples of raw fish dishes include *koi pla, pla som, pla jom,* and raw *pla ra*. Additionally, having a relative with bile duct cancer—such as a grandparent, father, mother, or sibling—is another important risk factor to consider in screening recommendations. Additionally, efforts focused on waste treatment cleanliness, health literacy, food safety, and ensuring food was free of parasites.^58^^,^^62^ Over 20 years (1989–2018), this comprehensive program reduced *O. viverrini* infection and CCA.^46^^,^^47^^,^^56^^,^^63^

The “Decade Strategy for Eliminating Opisthorchis and Cholangiocarcinoma (2016–2025)” guides prevention and control. It is supplemented by CCA screening, therapy, and prevention (Food Safety, School-Based Health Education, *O. viverrini* Screening)^62^ Furthermore, long-term parasite management diminished CCA.^41^ CCA continues to manifest, particularly in older persons exposed to risk factors during adolescence.^52^^,^^64^^,^^65^ A prolonged interval between *O. viverrini* infection and CCA diagnosis strongly correlates with a poorer prognosis.^66^

Thai cancer registries have greater than 90% coverage according to the standards of national and international organizations^67^^,^^68^; however, our analysis found insufficient morphological verification. This finding suggests that CCA is difficult to diagnose and requires improved testing. Further intensive training is needed to enhance detection capacities and more thorough reporting.

This study has several strengths and limitations. A strength of this study lies in being the first regional analysis of CCA trends and projections using several projection models and a 10-year population-based cancer registry dataset, including all four Thai regions. The short study period may have led to complications in the Nordpred model; however, the analytical procedure was adjusted. Although a robust technique, the projection assumes the continuation of present patterns and might fail to handle unforeseen developments. Furthermore, most samples originated from the northern and northeastern regions, whit the highest rates of CCA diagnosis. At the regional level, the central and southern regions had few samples. Specifically, for the central and southern regions, this small sample size might mean that our study could not determine definitive trends for those regions. Since this was the first nationwide study, the study areas were not fully representative, a weakness that should be addressed in future research.

This regional analysis of CCA trends in Thailand from 2012 to 2021 shows a declining incidence rate, with further decreases projected by 2026. However, regional disparities persisted, with the northeastern and northern regions maintaining high rates. The current study highlights the need for targeted interventions addressing region-specific risk factors, improved diagnostics, and research into emerging CCA risk factors to achieve more equitable health outcomes and sustained reduction in the national CCA burden.

## Contributors

Supot Kamsa-ard was the corresponding author of this study. Oraya Sahat was the principal author and, together with all authors, supported the overall study conception and design. Surin Uadrang, Atit Leklob, Wasan Chansaard, Nithima Sriket, Chalongpon Santong, and Karnchana Daoprasert curated the data. Surichai Bilheem oversaw data analysis with support from Apiradee Lim, Siriporn Kamsa-ard, and Apiporn Thinkhamrop Suwannatrai. All authors have reviewed the previous version of the manuscript and approved the final.

## Data sharing statement

The data used in this study contained personal information from four Thai population-based cancer registries; therefore, it cannot be made public to protect privacy. Data access requests must be addressed to relevant registries for review under strict ethical and statutory guidelines.

## Declaration of interests

The authors declare that they have no conflicts of interest.

## References

[1] London W.T., Petrick J.L., McGlynn K.A., Thun M., Linet M.S., Cerhan J.R., Haiman C.A., Schottenfeld D. (2017). Cancer Epidemiology and Prevention.

[2] Ferlay J., Ervik M., Lam F. (2024). Global cancer observatory: cancer today. https://gco.iarc.who.int/today.

[3] Lee T.Y., Lin J.T., Kuo K.N. (2013). A nationwide population-based study shows increasing incidence of cholangiocarcinoma. Hepatol Int.

[4] Alsaleh M., Leftley Z., Barbera T.A. (2019). Cholangiocarcinoma: a guide for the nonspecialist. Int J Gen Med.

[5] Florio A.A., Ferlay J., Znaor A. (2020). Global trends in intrahepatic and extrahepatic cholangiocarcinoma incidence from 1993 to 2012. Cancer.

[6] Khan S.A., Tavolari S., Brandi G. (2019). Cholangiocarcinoma: epidemiology and risk factors. Liver Int.

[7] Banales J.M., Marin J.J.G., Lamarca A. (2020). Cholangiocarcinoma 2020: the next horizon in mechanisms and management. Nat Rev Gastroenterol Hepatol.

[8] Rojanamatin J., Ukranum W., Supaattagorn P. (2021).

[9] Imsamran W., Pattatang A., Supaattagorn P. (2018).

[10] Imsamran W., Chaiwerawattana A., Wiangnon S. (2015).

[11] Kaewpitoon S.J., Rujirakul R., Joosiri A. (2016). GIS database and google map of the population at risk of cholangiocarcinoma in Mueang Yang District, Nakhon Ratchasima Province of Thailand. Asian Pac J Cancer Prev.

[12] Seeherunwong A., Chaiear N., Khuntikeo N., Ekpanyaskul C. (2022). The proportion of occupationally related cholangiocarcinoma: a tertiary hospital study in Northeastern Thailand. Cancers.

[13] Ito T., Sakurai-Yageta M., Goto A., Pairojkul C., Yongvanit P., Murakami Y. (2014). Genomic and transcriptional alterations of cholangiocarcinoma. J Hepatobiliary Pancreat Sci.

[14] Haswell-Elkins M.R., Mairiang E., Mairiang P. (1994). Cross-sectional study of Opisthorchis viverrini infection and cholangiocarcinoma in communities within a high-risk area in northeast Thailand. Int J Cancer.

[15] Kamsa-ard S., Kamsa-ard S., Luvira V., Suwanrungruang K., Vatanasapt P., Wiangnon S. (2018). Risk factors for cholangiocarcinoma in Thailand: a systematic review and meta-analysis. Asian Pac J Cancer Prev.

[16] Poomphakwaen K., Promthet S., Kamsa-Ard S. (2009). Risk factors for cholangiocarcinoma in Khon Kaen, Thailand: a nested case-control study. Asian Pac J Cancer Prev.

[17] Sithithaworn P., Andrews R.H., Nguyen V.D. (2012). The current status of opisthorchiasis and clonorchiasis in the Mekong Basin. Parasitol Int.

[18] Sripa B., Kaewkes S., Sithithaworn P. (2007). Liver fluke induces cholangiocarcinoma. PLoS Med.

[19] Khuntikeo N., Loilome W., Thinkhamrop B., Chamadol N., Yongvanit P. (2016). A comprehensive public health conceptual framework and strategy to effectively combat cholangiocarcinoma in Thailand. PLoS Negl Trop Dis.

[20] Honjo S., Srivatanakul P., Sriplung H. (2005). Genetic and environmental determinants of risk for cholangiocarcinoma via Opisthorchis viverrini in a densely infested area in Nakhon Phanom, northeast Thailand. Int J Cancer.

[21] Khuntikeo N., Chamadol N., Yongvanit P. (2015). Cohort profile: cholangiocarcinoma screening and care program (CASCAP). BMC Cancer.

[22] WHO (2013). WHO | international classification of diseases for oncology, 3rd ed. (ICD-O-3). http://www.who.int/classifications/icd/adaptations/oncology/en/.

[23] World Health Organization (2013). https://iris.who.int/handle/10665/96612.

[24] Office of the National Economic and Social Development Board (2013).

[25] Bray F., Colombet M., Aitken J.F. (2023). Cancer Incidence in Five Continents, Vol. XII (IARC CancerBase No. 19).

[26] Boyle P., Parkin D.M. (1991). Cancer registration: principles and methods. Statistical methods for registries. IARC Sci Publ.

[27] Kim H.J., Fay M.P., Feuer E.J., Midthune D.N. (2000). Permutation tests for joinpoint regression with applications to cancer rates. Stat Med.

[28] Moller B., Fekjaer H., Hakulinen T. (2003). Prediction of cancer incidence in the Nordic countries: empirical comparison of different approaches. Stat Med.

[29] Holford T.R. (1983). The estimation of age, period and cohort effects for vital rates. Biometrics.

[30] Holford T.R., Zhang Z., McKay L.A. (1994). Estimating age, period and cohort effects using the multistage model for cancer. Stat Med.

[31] Czajkowski M., Gill R., Rempala G. (2008). Model selection in logistic joinpoint regression with applications to analyzing cohort mortality patterns. Stat Med.

[32] Chen H.S., Portier K., Ghosh K. (2012). Predicting US- and state-level cancer counts for the current calendar year: Part I: evaluation of temporal projection methods for mortality. Cancer.

[33] Zhu L., Pickle L.W., Ghosh K. (2012). Predicting US- and state-level cancer counts for the current calendar year: Part II: evaluation of spatiotemporal projection methods for incidence. Cancer.

[34] Carstensen B. (2007). Age-period-cohort models for the Lexis diagram. Stat Med.

[35] Carstensen B., Plummer M., Laara E., Hills M. (2023). Epi: a package for statistical analysis in epidemiology. R package version 2.47.1. https://rdocumentation.org/packages/Epi/versions/2.47.1.

[36] Moller B., Fekjaer H., Hakulinen T. (2002). Prediction of cancer incidence in the Nordic countries up to the year 2020. Eur J Cancer Prev.

[37] Mistry M., Parkin D.M., Ahmad A.S., Sasieni P. (2011). Cancer incidence in the United Kingdom: projections to the year 2030. Br J Cancer.

[38] R Core Team (2023). https://www.R-project.org/.

[39] Posit team (2023). http://www.posit.co/.

[40] National Cancer Institute (2017). https://surveillance.cancer.gov/joinpoint/.

[41] Sriamporn S., Pisani P., Pipitgool V., Suwanrungruang K., Kamsa-ard S., Parkin D.M. (2004). Prevalence of Opisthorchis viverrini infection and incidence of cholangiocarcinoma in Khon Kaen, Northeast Thailand. Trop Med Int Health.

[42] Sripa B., Pairojkul C. (2008). Cholangiocarcinoma: lessons from Thailand. Curr Opin Gastroenterol.

[43] Chaiteerakij R., Pan-Ngum W., Poovorawan K., Soonthornworasiri N., Treeprasertsuk S., Phaosawasdi K. (2017). Characteristics and outcomes of cholangiocarcinoma by region in Thailand: a nationwide study. World J Gastroenterol.

[44] Yeesoonsang S., McNeil E., Virani S. (2018). Trends in incidence of two major subtypes of liver and bile duct cancer: hepatocellular carcinoma and cholangiocarcinoma in Songkhla, Southern Thailand, 1989-2030. J Cancer Epidemiol.

[45] Kamsa-Ard S., Luvira V., Pugkhem A. (2015). Association between praziquantel treatment and cholangiocarcinoma: a hospital-based matched case-control study. BMC Cancer.

[46] Kamsa-Ard S., Luvira V., Suwanrungruang K. (2019). Cholangiocarcinoma trends, incidence, and relative survival in Khon Kaen, Thailand from 1989 through 2013: a population-based cancer registry study. J Epidemiol.

[47] Kamsa-Ard S., Santong C., Kamsa-Ard S. (2021). Decreasing trends in cholangiocarcinoma incidence and relative survival in Khon Kaen, Thailand: an updated, inclusive, population-based cancer registry analysis for 1989-2018. PLoS One.

[48] Lin C.R., Lee Y.K., Chiang C.J., Yang Y.W., Chang H.C., You S.L. (2022). Secular trends of intrahepatic cholangiocarcinoma in a high endemic area: a population-based study. World J Gastroenterol.

[49] Saha S.K., Zhu A.X., Fuchs C.S., Brooks G.A. (2016). Forty-year trends in cholangiocarcinoma incidence in the U.S.: intrahepatic disease on the rise. Oncologist.

[50] Altekruse S.F., Petrick J.L., Rolin A.I. (2015). Geographic variation of intrahepatic cholangiocarcinoma, extrahepatic cholangiocarcinoma, and hepatocellular carcinoma in the United States. PLoS One.

[51] Sithithaworn P., Yongvanit P., Duenngai K., Kiatsopit N., Pairojkul C. (2014). Roles of liver fluke infection as risk factor for cholangiocarcinoma. J Hepatobiliary Pancreat Sci.

[52] Upatham E.S., Viyanant V., Kurathong S. (1984). Relationship between prevalence and intensity of Opisthorchis viverrini infection, and clinical symptoms and signs in a rural community in north-east Thailand. Bull World Health Organ.

[53] Banales J.M., Cardinale V., Carpino G. (2016). Expert consensus document: cholangiocarcinoma: current knowledge and future perspectives consensus statement from the European Network for the Study of Cholangiocarcinoma (ENS-CCA). Nat Rev Gastroenterol Hepatol.

[54] Global Burden of Disease Cancer C, Fitzmaurice C., Dicker D. (2015). The global burden of cancer 2013. JAMA Oncol.

[55] Pugkhem A., Kamsa-Ard S., Kamsa-Ard S., Luvira V., Luvira V., Bhudhisawasdi V. (2024). Pesticide exposure and risk of cholangiocarcinoma: a hospital-based matched case-control study. Trop Med Int Health.

[56] Jongsuksuntigul P., Imsomboon T. (2003). Opisthorchiasis control in Thailand. Acta Trop.

[57] Jongsuksuntigul P., Imsomboon T. (1998). Epidemiology of opisthorchiasis and national control program in Thailand. Southeast Asian J Trop Med Public Health.

[58] Bureau of Health Promotion (1987).

[59] Pinlaor S., Ma N., Hiraku Y. (2004). Repeated infection with Opisthorchis viverrini induces accumulation of 8-nitroguanine and 8-oxo-7,8-dihydro-2'-deoxyguanine in the bile duct of hamsters via inducible nitric oxide synthase. Carcinogenesis.

[60] Pinlaor S., Prakobwong S., Boonmars T., Wongkham C., Pinlaor P., Hiraku Y. (2009). Effect of praziquantel treatment on the expression of matrix metalloproteinases in relation to tissue resorption during fibrosis in hamsters with acute and chronic Opisthorchis viverrini infection. Acta Trop.

[61] Charoensuk L., Pinlaor P., Prakobwong S. (2011). Curcumin induces a nuclear factor-erythroid 2-related factor 2-driven response against oxidative and nitrative stress after praziquantel treatment in liver fluke-infected hamsters. Int J Parasitol.

[62] Khuntikeo N., Titapun A., Loilome W. (2018). Current perspectives on opisthorchiasis control and cholangiocarcinoma detection in Southeast Asia. Front Med.

[63] Kamsa-ard S., Wiangnon S., Suwanrungruang K. (2011). Trends in liver cancer incidence between 1985 and 2009, Khon Kaen, Thailand: cholangiocarcinoma. Asian Pac J Cancer Prev.

[64] Grundy-Warr C., Andrews R.H., Sithithaworn P. (2012). Raw attitudes, wetland cultures, life-cycles: socio-cultural dynamics relating to Opisthorchis viverrini in the Mekong Basin. Parasitol Int.

[65] Kaewpitoon N., Kaewpitoon S.J., Pengsaa P. (2008). Opisthorchiasis in Thailand: review and current status. World J Gastroenterol.

[66] Sithithaworn P., Haswell-Elkins M. (2003). Epidemiology of Opisthorchis viverrini. Acta Trop.

[67] Sirirungreung A., Buasom R., Jiraphongsa C., Sangrajrang S. (2018). Data reliability and coding completeness of cancer registry information using reabstracting method in the national cancer Institute: Thailand, 2012 to 2014. J Glob Oncol.

[68] Suwanrungruang K., Sriplung H., Attasara P. (2011). Quality of case ascertainment in cancer registries: a proposal for a virtual three-source capture-recapture technique. Asian Pac J Cancer Prev.

